# Brain-to-brain synchrony increased during interpersonal touch in romantic lovers: an EEG-based hyperscanning study

**DOI:** 10.1186/s40359-024-02051-7

**Published:** 2024-10-16

**Authors:** Chenghao Zhou, Xiaowei Jiang, Yanan Chen, Chunlei Ge, Na Ao, Feng Du

**Affiliations:** 1 Institute of Psychology and Behavior, Henan University, Kaifeng, 475001 China; 2 Institute of Cognition, Brain and Health, Henan University, Kaifeng, 475001 China; 3 Department of Psychology, New York University, New York, NY 10003 USA; 4 Department of Bioengineering, University of Pennsylvania, Philadelphia, PA 19104-6321 USA; 5 CAS Key Laboratory of Behavioral Science, Institute of Psychology, Chinese Academy of Sciences, Beijing, 100101 China; 6 Department of Psychology, University of Chinese Academy of Sciences, Beijing, 100101 China

**Keywords:** EEG-based hyperscanning, Interpersonal touch, Brain-to-brain synchrony, Romantic love, Dynamic interpersonal functional connectivity

## Abstract

**Background:**

Interpersonal touch is an essential element of human social life. It’s unclear whether the neural patterns of interpersonal touch are specific to intimate relationships or generally apply to other social relationships. Romantic lovers are typically intimate and have a high level of interpersonal touch. Currently, researchers focused on the neurobiological basis and neural processes of romantic love.

**Methods:**

110 participants finished two resting-state blocks, no-handholding and handholding conditions, with Electroencephalogram (EEG). We aimed to explore the differences in the brain-brain synchrony pattern of interpersonal touch between romantic lovers and strangers by calculating dynamic interpersonal functional connectivity (dIFC) via EEG-based hyperscanning.

**Results:**

Our results supported that the neural processing of interpersonal touch is a dynamic process. At first half, both groups tended to adapt, and then interpersonal touch increased the dIFC between romantic lovers and decreased the dIFC between strangers. Finally, we employed Support Vector Machine (SVM) to classify EEG signals into two different relationships. SVM recognized two relationships with an accuracy of 71% and 0.77 AUC of ROC at the first half, a 73% accuracy and 0.8 AUC of ROC at the second half.

**Conclusions:**

Our study indicates that interpersonal touch may have different meanings between romantic lovers and strangers. Specifically, interpersonal touch enhances the dIFC between romantic lovers while reducing the dIFC between strangers. The research has important implications for planning touch-based interventions in social and medical care.

## Introduction

Interpersonal touch during the COVID-19 pandemic was limited and compromised; however, it is the foundation of social connections and can benefit relationships. Sometimes, interpersonal touch is related to emotional communication. In intimate relationships, interpersonal touch from one’s partner can decrease the responses to electric shock [1], reduce stress [2], and relieve pain [3]. On the other hand, interpersonal touch among strangers who have no desire to build a relationship, such as accidentally bumping into each other on a crowded subway or a pat-down at an airport security check, produces a host of negative consequences [4]. Therefore, it is urgent to explore the effect of interpersonal touch in intimate relationships and between strangers to elucidate the neurocognitive mechanism of interpersonal touch further. In the present study, in addition to investigating the neural pattern of interpersonal touch, we examine the influence of interpersonal touch on varying types of relationships. For example, in intimate partners, hugs and holding hands can relieve and reduce partners’ suffering. For strangers, barriers such as distance between each other might provide a sense of safety and relaxation.

### Interpersonal touch

Interpersonal touch is the basis of human communication. Accumulating evidence suggests that interpersonal touch is essential in social relationships and plays an important role in socioemotional, cognitive, and neural development [5, 6]; it affects sensory perception [7, 8], and benefits the physical and mental health of adults in close relationships, such as romantic relationships and friendships [1, 9]. Studies have shown that social contact affects emotions and reduces sadness and pain [10–12]. Researchers have also reported that skin-to-skin contact may have analgesic effects on human infants undergoing minor medical procedures [13] and therapeutic effects, reducing pain in patients with cancer or chronic pain [14]. Lack of interpersonal touch or physical neglect can lead to a higher level of aggression in adolescents [15]. Long-term deprivation of interpersonal touch affects all aspects of health and is associated with increased stress, anxiety and depression [16].

In the past few decades, numerous studies have confirmed the positive effects of interpersonal touch. Fisher and his colleagues arranged a chance contact between participants and staff in a library and then compared participant responses with noncontact conditions. Participants were surveyed about their emotional state and feelings toward the library clerk and the library environment. The results showed that participants in the contact condition responded more positively than participants in the noncontact condition [17]. Other researchers have examined how interpersonal touch affected the tips earned in a restaurant context [18–20]. They found that customers provided a higher tip when physical contact between the waiter and the customer occurred than without physical contact [18, 19]. These studies also evaluated the effect of sex but obtained different results. Some researchers found that male customers gave higher tips [18], while others found that female customers tipped more, especially when waiters were more attractive [21].

These early studies were mainly questionnaire research and field studies. In recent years, relevant studies on physiological and psychological responses have found that interpersonal touch has beneficial effects on adverse life events. Interpersonal touch is thought to buffer stress and play a key regulatory role in response to acute stressors, including cortisol level and heart rate changes, ultimately promoting social bonding [22, 23]. Unsurprisingly, the benefits and importance of interpersonal touch in interpersonal communication were demonstrated in romantic relationships [24, 25]. However, the neural pattern of interpersonal touch in different interpersonal relations is still not well understood, especially in romantic lovers and strangers.

### Interpersonal touch in different interpersonal relations

Romantic love is generally regarded as one of the most profound and meaningful human emotions and plays an essential role in human reproduction, development, evolution, and survival [26]. Romantic relationships are typically intimate and have high levels of interpersonal touch; this interpersonal touch improves the quality of nonverbal physical communication. Recent research has shown that romantic partners elicit more physiological coupling during cooperative tasks than those completed by friends and strangers and that this increase in physiological coupling is also correlated with their task performance [25].

Studies have shown that the effect of interpersonal touch depends on the identity of the interaction partner; compared with a stranger, holding a partner’s hand reduces anxiety and blood pressure in response to stress, as well as activating areas of the brain that support emotional and behavioral threat responses [27, 28]. For instance, holding hands with their husbands relieves wives’ pain during labor, and massage and breath coaching from husbands decreases depressed mood, anxiety, pain, and postpartum depression in wives and shortens their labors and length of hospital stays [29].

A study by Goldstein et al. (2017) [30] reported that touch increases interpersonal physiological coupling during the experience of pain. They recruited 22 pairs of romantic lovers; these couples were assigned to the pain receiver or pain observer roles under pain/no-pain and touch/no-touch conditions, and participants’ electrocardiogram (ECG) and respiratory rates were recorded. The results showed that partner touch increased interpersonal respiratory coupling in both pain and no-pain conditions and increased heart rate coupling in pain conditions. In addition, physiological coupling was diminished by pain in the absence of the partner’s touch [30]. Similarly, another study by Goldstein et al. (2018) [3] recorded EEG to examine brain-to-brain coupling during the experience of pain with interpersonal touch. The results suggested that holding hands increased the brain-to-brain coupling of partners, which reduced pain. The brain-to-brain coupling network mainly involved the somatosensory cortex (according to the painful stimulus’s location) and the observer’s right hemisphere [3]. In 2021, Long et al. (2021) [31] used functional near-infrared spectroscopy-based hyperscanning to measure the brain-to-brain synchrony between romantic lovers and between friends. They recruited 22 romantic lovers and 22 opposite-sex friends; each pair completed a task while holding hands or with vocal communication during the experiment. The results showed that a significantly higher level of synchronization was achieved when romantic lovers held hands without speaking than when they did not hold hands but talked with each otherl. However, the opposite pattern was observed in pairs of opposite-sex friends, with brain activity significantly more synchronized during vocal communication than while holding hands.

## The brain-to-brain synchrony

Recent research on interpersonal touch has underscored its significant impact on social and emotional well-being. To test our hypothesis regarding the unique neural dynamics of interpersonal touch, a method that simultaneously records the brain activity of two individuals is required—something that cannot be achieved through single-brain approaches. Hyperscanning has been used to explore the neural underpinnings of human communication between two or more interacting brains [32, 33]. Interpersonal touch, such as handholding or hugging, has been shown to reduce stress, alleviate pain, and enhance social bonds, particularly in romantic relationships. Studies have also demonstrated that the neural mechanisms underlying these effects differ between relationship types, with romantic partners showing increased physiological and neural synchrony during touch compared to strangers.

Interpersonal Functional connectivity (IFC), a key concept in brain-to-brain synchrony, includes both static functional connectivity (sFC) and dynamic interpersonal functional connectivity (dIFC). These are quantified through correlation, covariance, and mutual information of time series from different brain regions [34]. While sFC analyzes the entire time series of brain signals as a single snapshot, dFC examines changes in these connections over time, making it more sensitive to the dynamic nature of brain activity. This dynamic perspective is crucial for studying processes like cognition and emotion, which are inherently variable over time.

Magnetic resonance imaging(MRI)has high spatial resolution and can collect both functional and structural images, which can help researchers estimate FC, structural connections, and their relationships based on high spatial resolution data [35, 36]. However, fMRI’s low temporal resolution (typically around 2 s) limits its ability to capture the rapid, time-varying characteristics of neural activity or to reflect the dynamic nature of human communication. To address these limitations and provide a more nuanced understanding of functional connectivity, researchers have increasingly focused on dFC. Unlike sFC, dFC captures how brain connections fluctuate over time, providing insights into the temporal dynamics of neural processes [25, 37, 38]. Electroencephalography (EEG), which records the spontaneous, rhythmic electrical activity of neuronal groups via electrodes, offers several advantages: it is easy to use, noninvasive, and has high temporal resolution (typically 500-20k Hz). While most previous studies have relied on fMRI and functional near-infrared spectroscopy (fNIRS) to explore brain-to-brain synchrony (BtBS), these methods lack the temporal precision needed to observe rapid neural changes during interpersonal touch. Our study addresses this gap by utilizing EEG-based hyperscanning to capture real-time dFC between brains. By focusing on romantic partners and strangers, we provide new insights into how the context of a relationship modulates the neural effects of interpersonal touch.

Recent studies have shown promising results in this field. For example, Allen et al., (2018) [39] demonstrated the feasibility and replicability of dFC on an eyes-open and eyes-closed dataset. Shamsi used dFC as feature extraction to classify motor tasks, while Liu et al., (2019) [40] used dFC, by sorting the multiband static network from time sequence to classify emotion. Despite the potential of dFC, it also presents unique challenges in data analysis. The most frequently used method to analyze dFC was the sliding window method [41]. The sliding-window correlations method (SWC) is based on time-domain data analysis, it involves segmenting the time process into a set of time windows from the brain position (brain voxel or region) and calculating its paired-FC within the sliding window. SWC provides a direct measure of FC in the sliding window framework [42]. The following analysis of dynamic interpersonal functional connectivity of the present study was based on the sliding-window correlations method.

## The current study

Here, we hypothesize that brain-to-brain synchrony differs between romantic lovers and between opposite-sex strangers during interpersonal touch. Goldstein et al., (2018) [3] reported that interpersonal touch increased dynamic interpersonal functional connectivity (dIFC) between romantic lovers when one experienced pain. Another recent study showed that the dFC increase during interpersonal touch was larger than during vocal communication between romantic lovers, but similar patterns were not found in pairs of friends [31]. The relationship between romantic lovers is clearly defined, while the relationship between opposite-sex friends is fuzzy because they may develop into a couple. That is, opposite-sex friends may not be the appropriate comparison for romantic lovers unless the controls and screening method are rigorous.

We designed an EEG hyperscanning experiment to determine whether the brain-to-brain synchrony between romantic lovers differs from opposite-sex strangers during interpersonal touch. This study examined changes in the dIFC (via EEG) between heterosexual romantic lovers and opposite-sex strangers during touch/no-touch conditions in a naturalistic context to test the above hypotheses. Support Vector Machine (SVM) is then used as the classifier for two conditions as the traditional analysis methods ANOVA assumes that each connection between channels is independent and linear. SVM can also be extended to solve nonlinear classification tasks when the set of samples cannot be separated linearly [43]. Unlike previous research, such as Goldstein et al. (2017) [30] and Long et al. (2021) [31], which primarily focused on physiological coupling during static conditions or distinct interaction tasks, this study employs EEG-based hyperscanning to explore the temporal dynamics of brain-to-brain synchrony during interpersonal touch. By examining these dynamics between romantic partners and strangers, our approach offers novel insights into the real-time neural mechanisms underlying social touch, capturing aspects of brain activity that were not addressed in earlier studies. This methodological innovation allows for a more nuanced understanding of how neural synchrony evolves in different social contexts, particularly in relation to the unique qualities of interpersonal touch.

We predicted that interpersonal touch between romantic lovers would incite a significantly higher level of brain-to-brain synchronization than that between strangers. Interpersonal touch may increase the brain-to-brain synchrony of romantic lovers while decreasing the brain-to-brain synchrony of strangers. Interpersonal touch may also display differences in dIFC depending on relationship type.

## Materials and methods

### Participants

A total of 110 dextromanual, healthy undergraduates participated in the experiment (27 paired romantic lovers and 28 paired matched strangers, half male and half female). Table 1 shows the inclusion criteria of the subjects. We recruited the subjects through the internet, and they were double-checked before the experiment to see if they met the inclusion criteria requirements. Due to unexpected situations, as shown in Table 2, there were valid EEG-paired data for 78 people (18 pairs of romantic lovers and 21 pairs of strangers) and valid questionnaire data for 73 people among valid EEG-paired data (1 pair of romantic lovers and 4 strangers’ original data were numbered error and 1 stranger’s original data were missing). The sample size was determined based on considerations for previous study and priori power analysis. For example, Long (2021) [31] included twenty-two pairs of heterosexual romantic couples and 22 pairs of friends of opposite sexes, suggesting that our sample size is comparable to similar studies. Also, a priori power analysis was performed using G*Power Version 3.0.1 [44]. The power analysis performed for a repeated measures analysis of variance indicated that a sample size of 56 would detect an effect size of 0.20 for the Relationship Type × Hand Type interaction and achieve a power of 0.95 at an alpha level of 0.05. Given that the data in this study was paired data, paired data was not available once there is a problem with a person’s data, thus, our actual sample size exceeds the target priori sample size.

**Table 1:** The inclusion criteria of subjects

Type	Inclusion criteria
Couples	①Make sure the partner (heterosexuality) is the only one who has been in love with you for more than three months and less than two years
	②Physical and mental health, no obvious disability or physical disorder, no perm hair coloring during past three months
	③No claustrophobia or extreme fear of the dark
	④In this experiment, there was some physical contact between the partners
	⑤Wash your hair (to better exfoliate/keep your scalp clean) before this experiment
Strangers	①Keep single for more than one year, no relationship experience
	②Healthy in body and mind, with no obvious disability or physical impairment
	③No claustrophobia or extreme fear of the dark
	④In this experiment, there was some physical contact between the group
	⑤Wash your hair (to better exfoliate/keep your scalp clean)
	⑥Two people who know each other (opposite sex) cannot register at the same time, people with the same major will not be matched together

**Table 2:** Exclusion during the experiment

type	Exclusion	Count
Couples	The boy did not follow the experimental requirements	1
	one girl suspects her boyfriend of cheating	1
	the electrode cap has an issue	2
	< three months during the experiment	1
	felt sick during the experiment	2
	Electrode series	3
Strangers	Boy’s single time is shorter than three months	1
	felt sick during the experiment	2
	the electrode cap has an issue	2
	Electrode series	5

This study is conducted according to the Declaration of Helsinki. The research protocol has been approved by the Ethical Review of Psychological Research in Henan Province Key Laboratory of Psychology and Behavior. All participants will be clearly informed, and their consent will be obtained before collecting any data. Before the experiment, the subjects needed to reconfirm whether they met the inclusion requirements, and the experiment was carried out after signing the informed consent letter.

### Subjective measurements

We included Self-Rating Anxiety Scale (SAS) and Self-Rating Depression Scale (SDS) to ensure the participants’ mood state is relatively relaxed in recent and will not bias the experiment. We use a Two-way ANOVA to analyze gender and relationship type effects on the self-rated mental health scale (SAS and SDS).

The Self-Rating Depression Scale (SDS) includes 20 items on a four-point scale with occasionally or never = 1, sometimes = 2, often = 3 and continuous = 4. After recoding reversed items (2, 5, 6, 11, 12, 14, 16, 17, 18, 20 ), the final score equals forward scoring plus reverse scoring [45, 46]. In this study, the α = 0.793/ ω = 0.803 [47, 48].

The Self-Rating Anxiety Scale (SAS) includes 20 items on a four-point scale with occasionally or never = 1, sometimes = 2, often = 3 and continuous = 4. After recoding reversed items (5、9、13、17、19 ), the final score equals forward scoring plus reverse scoring [49]. In this study, the α = 0.837 / ω = 0.855 [47, 48].

## EEG recording and data acquisition

**Fig. 1:**
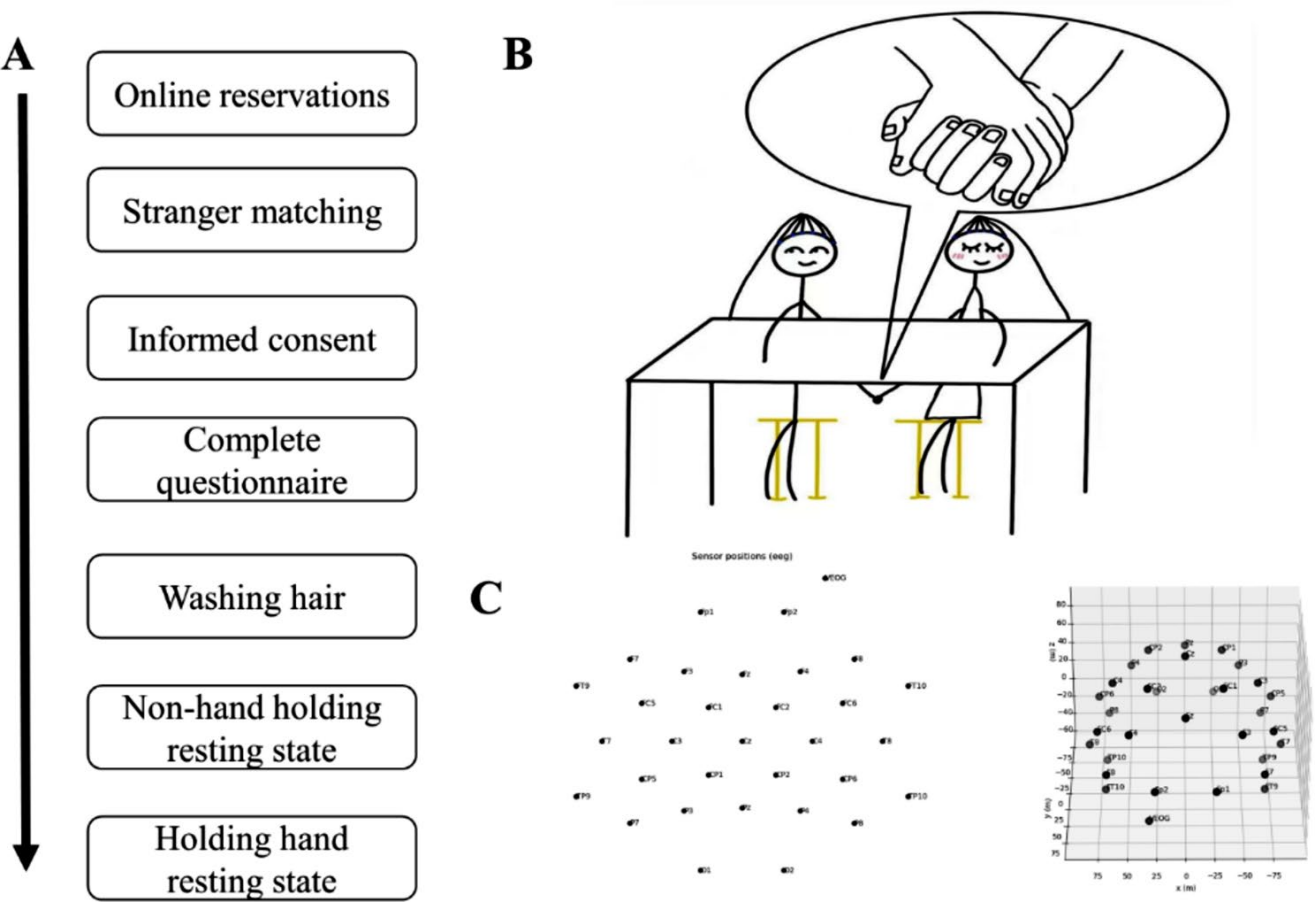
EEG Recording and Data Acquisition. **(A)** The experimental process during subject selection and data collection. **(B)** The laboratory environment and the subject environment. Here showed the hand-on condition, in hand-off condition, each participants put their hands flat on their thighs. **(C)** The electrodes’ position is according to the international 10–20 lead system

The experimental process is shown in Fig. 1A. Both romantic lovers and strangers needed to wash their hair independently after signing the informed consent letter and then performed two blocks in turn; no hand and handholding conditions. Each block collected 4-minute resting EEG signals. The whole EEG signal process was recorded to ensure that the subjects followed the experimenter’s instructions. This experiment was designed as a hyperscanning experiment. Figure 1B shows the laboratory environment and the subject environment. Before the formal experiment, we trained the participants to hold hands with fingers interlocked so that there would be consistency in each block. The neuroelectric hyperscanning recordings were performed with two 32-channel EEG acquisition systems (Males seated on the left with BrainAmp Live Cap 32, Brain Products, Germany, while females seated on the right with BrainAmp BrainCap 32, Brain Products, Germany - for each subject: 31 EEG + 1EOG electrodes placed according to the international 10–20 lead system, as shown in Fig. 1C). The impedances were maintained below 20 kOhm. The EEG/EOG signals were collected at a sampling frequency of 1000 Hz and filtered using a high-pass filter with a cutoff of 0.016 Hz and a lowpass with a cutoff of 500.0 Hz.

The reason why we haven’t counterbalanced the hand condition as follows: The first was to avoid potential carryover effects that could impact brain activity measurements. If the handholding condition was performed first, it could establish a neural baseline that might persist and influence the results of the subsequent no-handholding condition, leading to confounded findings. Secondly, considering that touch may transmit emotions and regulate emotions [8], to avoid the continuation effect of emotions affecting the subsequent experimental results, we putted the no-hand condition as a baseline condition for control in front of the handholding condition.

### EEG-source preprocessing and analysis method

**Fig. 2:**
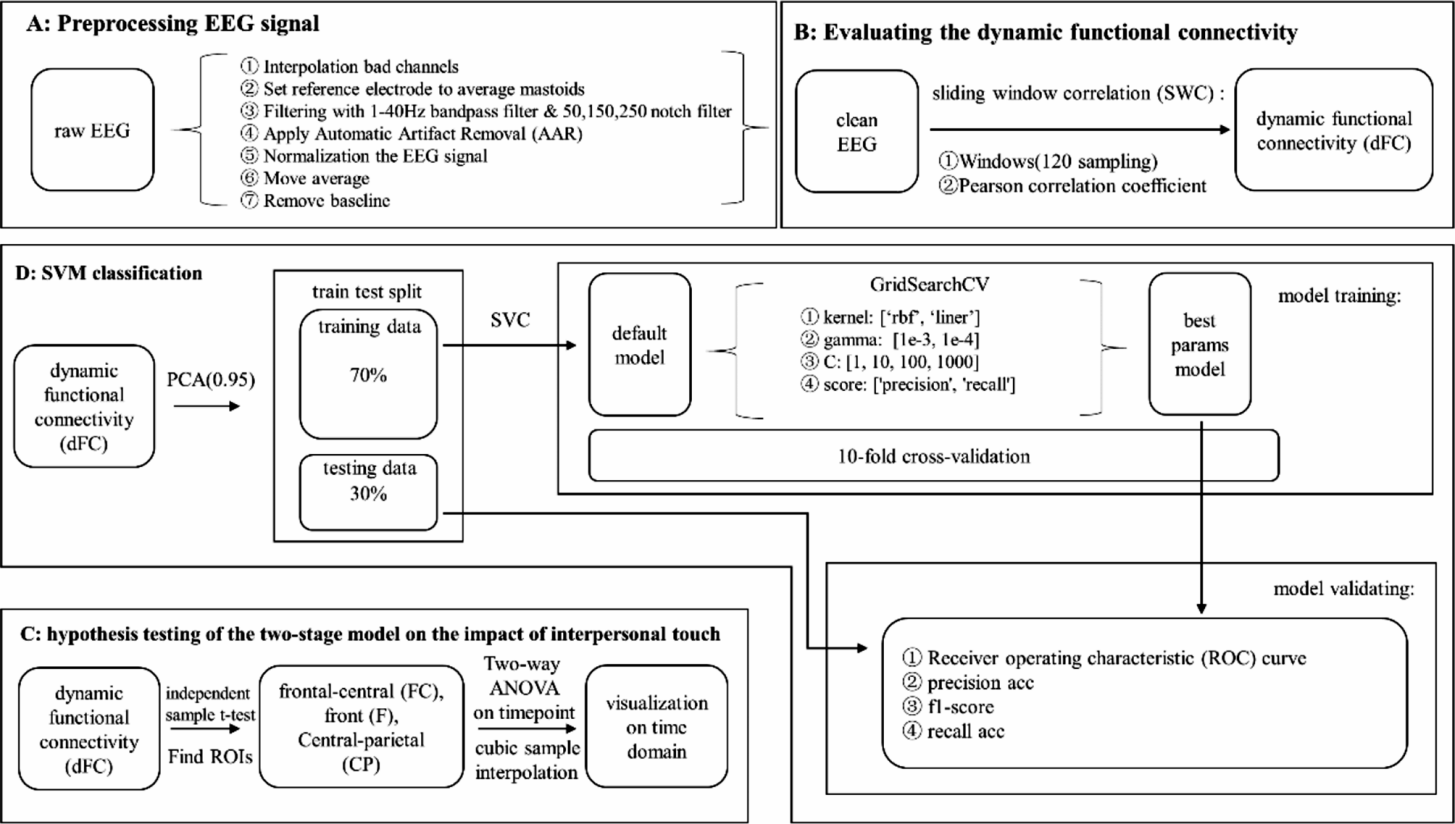
EEG-source preprocessing and analysis pipeline

The data preprocessing was based on EEGLAB 2020 [50], and further analysis of the EEG signal was carried out with MATLAB 2020a and Sklearn 0.23.2 [51] with Python 3.9. Figure 2 shows the flow of our preprocessing and analysis pipeline.

### Preprocessing EEG signal

Figure 2A shows the steps of the EEG signal preprocessing pipeline as follows:

Interpolation: Spherical linear interpolation was performed on bad channels based on the correlations between channels (lager than 0.95). Equation 1 shows the principle of spherical linear interpolation.

Equation 1 Exclusion during the experiment

$$\:\text{S}\text{l}\text{e}\text{r}\text{p}\left({p}_{0},{p}_{1};\text{t}\right)=\frac{\text{sin}\left[(1-t){\Omega\:}\right]}{\text{sin}{\Omega\:}}{p}_{0}+\frac{\text{sin}\left[t{\Omega\:}\right]}{\text{sin}{\Omega\:}}{p}_{1}$$

Set the reference electrode: We transferred the data with reference to the average mastoids (TP9 and TP10) in the 10–20 lead system. The average of the two mastoids is often used as an offline reference, averaging across the left and right mastoids and, furthermore, offering an asymmetric reference that should not prefer one of the hemispheres [52].

Filtering: The signal was first applied to a 50, 150, and 250 notch filter (± 2 Hz) and then filtered into 1–40 Hz filters with a bandpass filter.

Independent component analysis (ICA): We used the AAR plug-in for EEGLAB to automatically correct ocular and muscular artifacts in the EEG [53, 54].

Normalization: We normalized the power of each channel to transform it into a dimensionless expression so that we could compare subjects and different devices. This work used the zero mean unit variance (ZMUV) method to normalize the preprocessed EEG signal and locate it in the zero to one joint range. **Equation 2** shows the method to calculate the normalized signal for one channel.

### Equation 2 zero mean unit variance (ZMUV) normalization

$$\:{x}^{*}=\frac{x-min}{max-min}$$

Move average: Since we collected 4 min of resting-state EEG data in each block, we used move-average to improve signal quality and make computing more accessible and faster.

Remove baseline: Since the brain networks do not sit idle during the resting state, we use 500ms as a baseline to see the trend of change during the task.

### Evaluating the dynamic functional connectivity

Figure 2B shows the steps of the estimation of Functional Brain Connectivity as follows:

Estimation of Functional Brain Connectivity: From the perspective of the time domain, neural coupling indicators are adopted, given the uniqueness of the hyperscanning paradigm. This study will extract data features based on the Pearson correlation coefficient. The Pearson correlation coefficient r is a commonly used and easy-to-compute neural coupling index [55–57].

In dynamic interpersonal functional connectivity (dIFC) calculation, we used a 120 sampling size moving window [42, 58]. In each window, we followed the sliding window correlation (SWC) to calculate the sFC, which is the most commonly used method to characterize dIFC in neuroimages [42, 59].

### Hypothesis testing of the two-stage model on the impact of interpersonal touch

In this study, Regions of Interest (ROIs) were initially determined by identifying channels with significant voltage magnitude differences across the scalp, providing a two independent sample t-test to locate areas with notable neural activity changes (see Fig. 2C). While dynamic interpersonal functional connectivity (dIFC) was computed across the whole brain, our analysis specifically emphasized the differences in interpersonal functional connectivity between channels that exhibited significant voltage differences. Then we applied two-way mixed ANOVA (relationship factor and hand factor) on each timepoint of each channel (the correlation coefficient between an electrode of male and an electrode of female between each pair of participants). At last, we applied the False Discovery Rate (FDR) to prevent false positives [60]. For visualization, we used cubic sample interpolation to fit the trend of dynamic functional connections.

### SVM classification on dIFC

Support vector machine (SVM) has been widely used in EEG signal classification because of its convexity optimization and good generalization performance for high dimensional data [61, 62]. This study used SVM to predict the relationship types, using all timepoints as samples and each channel as a feature for prediction. In the classification model training, “romantic lovers” (or “1”) and “strangers” (or “0”) were set as predictive variables in this study.

The dataset is randomly shuffled and divided into two parts. The first part is the training dataset (70) to train the machine learning algorithm. The other part is the test dataset (30%), which acts as an independent test set to validate the final model. This study used 10-fold cross-validation (10-fold CV) to establish the training dataset classification model. More precisely, randomly selected 90% training dataset to train the model while the other 10% training dataset was used to verify it. The grid method is used to reduce the variance as a method to find the optimal parameters.

## Results

### Demographic variable

Artifact-free 240-s epochs were obtained in 78 out of 110 healthy subjects, as Table 3 shows the descriptive results of participants’ anxiety and depression score. The ANOVA results found no statistical significance for these two factors in both SDS: relationship type (*p* = 0.082, *F*(1,71) = 3.110, *η*^*2*^_*p*_ = 0.042) and gender (*p* = 0.102, *F*(1,71) = 2.750, *η*^*2*^_*p*_ = 0.037). The ANOVA results also found no statistical significance for these two factors in both SAS: relationship type (*p* = 0.230, *F*(1,71) = 1.465, *η*^*2*^_*p*_ = 0.020) and gender (*p* = 0.944, *F*(1,71) = 0.005, *η*^*2*^_*p*_ = 0.001).

**Table 3:** Descriptive statistics of SDS and SAS

			SDS	SAS
Type	Gender	N	Mean(SD)	Mean(SD)
Couples	Female	18	39.653(10.153)	49.792(11.680)
	Male	18	45.278(11.620)	50.208(8.383)
	Total	36	42.465(11.126)	50.000(10.022)
Strangers	Female	19	37.105(6.860)	47.237(9.883)
	Male	18	39.375(8.883)	46.458(8.904)
	Total	37	38.210(7.881)	46.858(9.297)
Total	Female	37	38.345(8.595)	48.480(10.721)
	Male	36	42.326(10.624)	48.333(8.732)
	Total	73	40.308(9.789)	48.408(9.723)

### Region of interest (ROI)

**Fig. 3:**
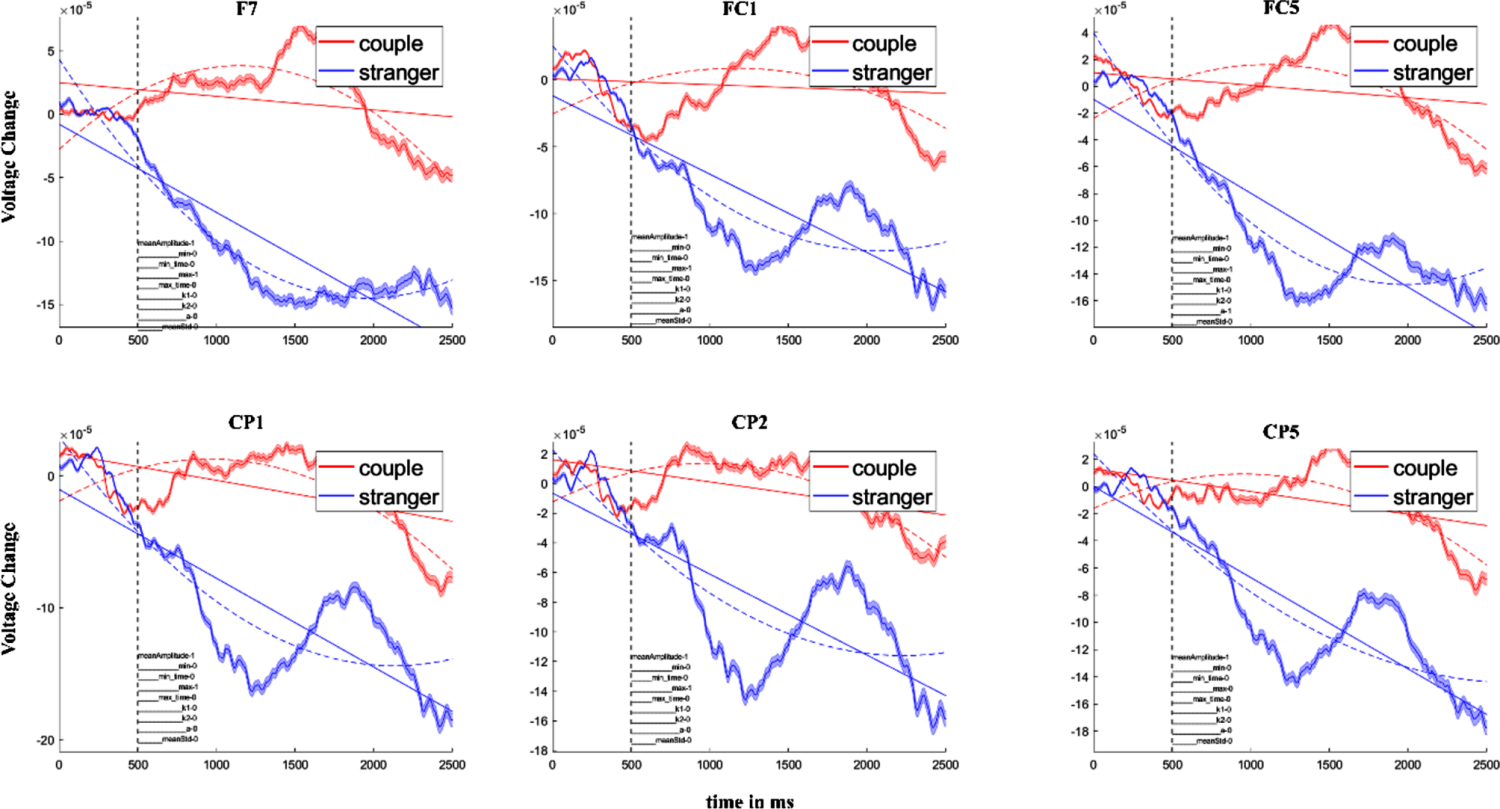
Brain results of voltage magnitude in relationship domain. We averaged the hands-on condition and hands-off condition for two relationship types (romantic lovers and strangers)

We found some significant differences in the frontal-central (FC1, *p* = 0.0197, *t* = 2.9626, FC5, *p* = 0.0113, *t* = 3.5133), central-parietal (CP1, *p* = 0.0197, *t* = 3.0153, CP2, *p* = 0.0325, *t* = 2.6012, CP5, *p* = 0.0320, *t* = 2.6469), and frontal (F7, *p* = 0.0113, *t* = 2.8504) in relation domains (relationship types between romantic lovers and strangers), as shown in Fig. 3. However, there are no significant differences in the hand domain (hands-on or hand-off). The ROI definition focused on the relationship effect because our primary goal was to distinguish brain activity patterns between romantic lovers and strangers. The subsequent analysis examined the interaction effects between relationship and touch conditions (handholding vs. no-handholding) to understand how these conditions jointly affect brain-to-brain synchrony.

### Dynamic interpersonal function connection(dIFC)

**Fig. 4:**
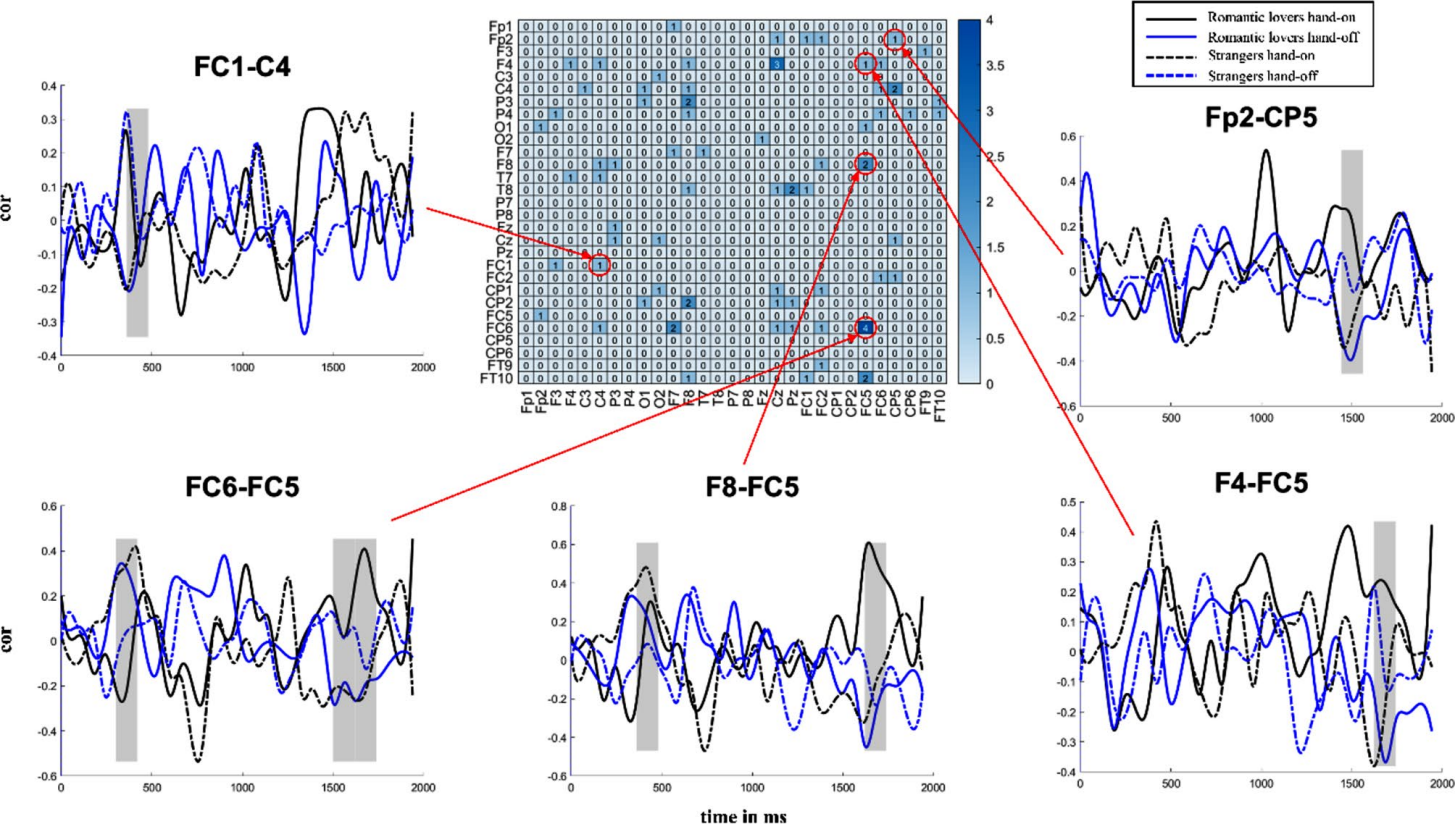
Brain results of dIFC. The heatmap shows the interaction results of ANOVA. The heatmap value shows the sum of timepoints with significant differences (the interaction between relationship and handholding factors) for the current channel. Red circles on the heatmap point out the channels that have significant differences among ROIs, which were directed by the demonstrations of dIFC on time domain. The red rectangles show the significant windows, which have been transformed into timepoints. Cubic sample interpolation was applied to fit the trend of dIFC

We present the results based on brain coupling derived from the whole time series of resting-state EEG and corresponding results derived from brain connectivity estimated from dynamic interpersonal functional connectivity (dIFC) in Fig. 4.

Figure 4 shows the heatmap of the interaction between relationship and hand condition, and shows the example of ROIs of the interaction of relationship and hand condition. The specific statistical results are as follows in different timepoint (TP) on different channels: FC1-C4(tp7: *p* = 0.0089, *F*(1,37) = 16.2146, *η*^*2*^_*p*_ = 0.3047), F4-FC5(tp28: *p* = 0.0217, *F*(1,37) = 13.8441, *η*^*2*^_*p*_ = 0.2723), F8-FC5(tp7: *p* = 0.0166, *F*(1,37) = 12.751, *η*^*2*^_*p*_ = 0.2563, tp28: *p* = 0.0058, *F*(1,37) = 24.2704, *η*^*2*^_*p*_ = 0.3961), FC6-FC5(tp6: *p* = 0.0433, *F*(1,37) = 10.1845, *η*^*2*^_*p*_ = 0.2158, tp7: *p* = 0.0425, *F*(1,37) = 12.1350, *η*^*2*^_*p*_ = 0.2470, tp26: *p* = 0.0433, *F*(1,37) = 8.8014, *η*^*2*^_*p*_ = 0.1922, tp28: *p* = 0.0433, *F*(1,37) = 9.2212, *η*^*2*^_*p*_ = 0.1995), FP2-CP5(tp25:*p* = 0.0328, *F*(1,37) = 12.7884, *η*^*2*^_*p*_ = 0.2569).

Here, the results reported outside the predefined ROIs. Our approach was to include results where at least one of the channels was part of the predefined ROIs, such as in the case of FC1-C4, where FC1 was within the initially selected ROIs, but C4 was not. This method allowed us to explore the functional connectivity between the predefined ROIs and other related channels to provide a broader understanding of the connectivity patterns.

As Fig. 4 shows, at tp7, the dIFC of F8-FC5, FC6-FC5 shows a significant difference on the interaction effect (type*hand on/off). On that point, the functional connectivity of hands-off is larger than the functional connectivity of hands-on between romantic lovers, while the functional connectivity of hands-on is larger than that of hands-off between strangers. However, the functional connectivity of FC1-C4 at tp7 shows that hands-on condition is larger than hands-off condition between romantic lovers while hands-off condition is larger than hands-on condition between strangers. At tp25, the functional connectivity of FP2-CP5 shows that hands-on condition is larger than hands-off condition between romantic lovers while hands-off condition is larger than hands-on condition between strangers. The tp26 of FC6-FC5 and the tp28 of F4-FC5, F8-FC5 and FC6-FC5 have the same tendency as FP2-CP5.

### SVM model results

**Fig. 5:**
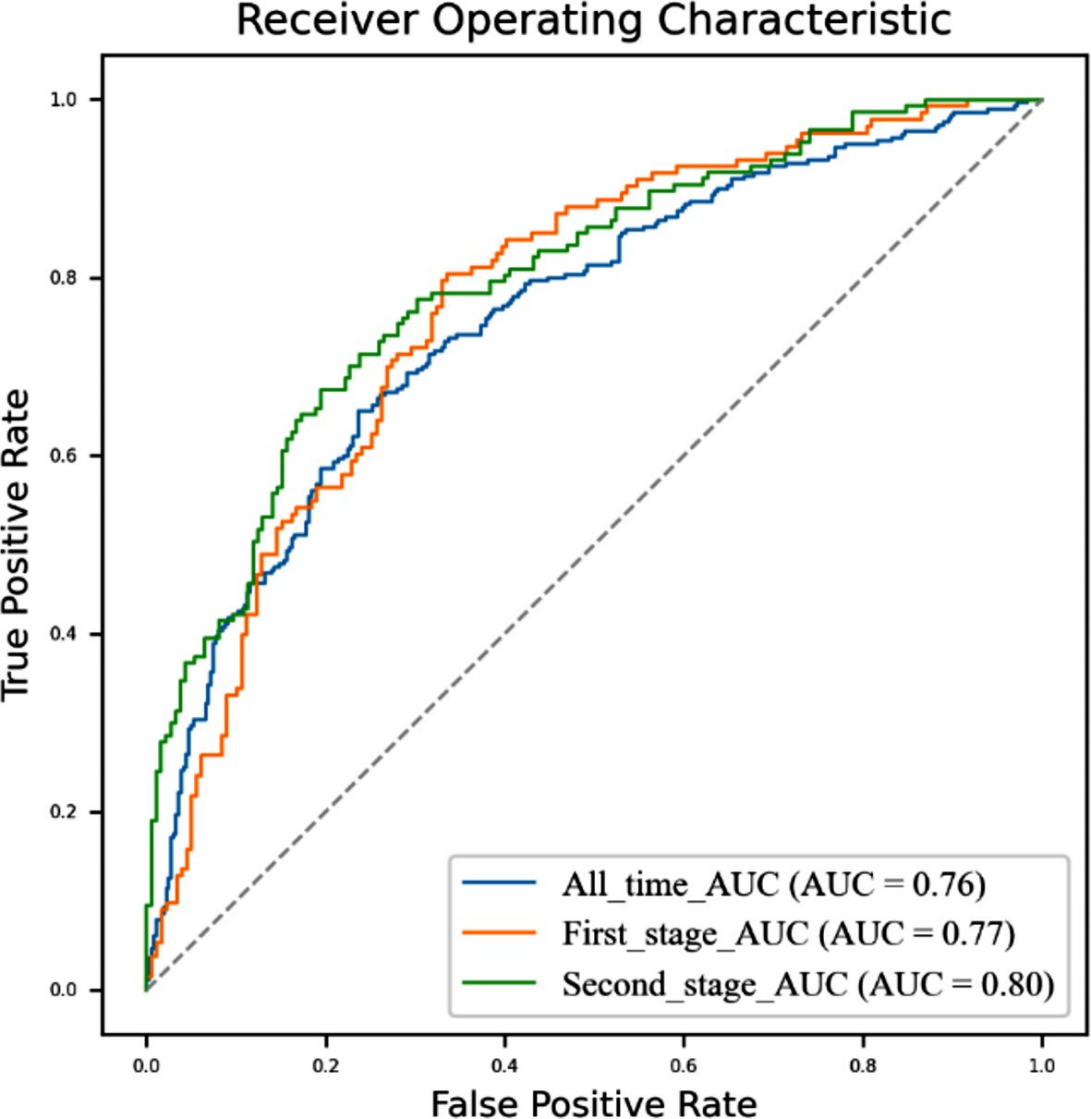
The ROC curve and AUC of each stage’s model by the SVM model

As shown in Table 4, combining the precision, recall rate, and F1 index of the test dataset, as well as the ROC curve and AUC of each half’s model (Fig. 5), the second half had a better performance than the first half. The model results were provided by the Scikit-Learning software package based on Python 3.9 [51]. The precision of the first half can reach about 71%, while F1, recall rate, and accuracy are above 70%, and the AUC of ROC reaches 0.77. The precision of the second half can reach about 73%, while F1, recall rate, and accuracy are above 70%, and the AUC of ROC reaches 0.80. These results suggest that the classification model established by the SVM was robust, but we do not claim the differences between the first and second halves to be statistically significant.

**Table 4:** Detailed classification report

type		precision	recall	f1-score	support
All	0	0.73	0.76	0.75	364
	1	0.67	0.64	0.65	280
	accuracy			0.71	644
	macro avg	0.70	0.70	0.70	644
	weighted avg	0.71	0.71	0.71	644
First stage	0	0.77	0.72	0.74	179
	1	0.65	0.71	0.68	133
	accuracy			0.71	312
	macro avg	0.71	0.71	0.71	312
	weighted avg	0.72	0.71	0.72	312
Second stage	0	0.78	0.73	0.75	185
	1	0.68	0.73	0.71	147
	accuracy			0.73	332
	macro avg	0.73	0.73	0.73	332
	weighted avg	0.73	0.73	0.73	332

0 = strangers; 1 = romantic lovers

## Discussion

We examined the brain-to-brain synchrony during interpersonal touch between romantic lovers and between opposite-sex strangers in a naturalistic resting-state task. We found that in the early stage (TP7), holding hands was stressful for both romantic lovers and strangers, which caused the brain-to-brain synchrony fluctuate from channel to channel in both two group. However, the results were more consistent in the late stage (TP26 and TP28). SVM results suggested that Stage two was better than Stage one at distinguishing the differences in brain coupling between the lovers and the strangers. The results suggested that the result of holding hands differed between the two groups. As the romantic lovers adjusted to holding hands, their brain-to-brain synchrony became more consistent, and holding hands boosted brain-to-brain synchrony. This difference between romantic lovers and strangers may be due to the tension or discomfort caused by physical contact with a stranger. Holding hands may lead to decrease brain-to-brain synchrony. Brain-to-brain synchronization results between lovers were found to be significantly higher than between strangers, which is consistent with brain-to-brain synchronization findings from another interpersonal touch study [31] and a study involving a button-press cooperation task [25].

The present study collected EEG data during interpersonal touch/no-touch conditions between romantic lovers and between strangers. The results suggested that dynamic interpersonal functional connectivity (dIFC) could distinguish between romantic lovers and strangers. After a chaotic adaptation, romantic lovers showed higher functional connectivity than strangers. Our study may help explain the neurocognitive pattern of interpersonal touch between romantic lovers and between opposite-sex strangers during a naturalistic resting context as opposed to other forms of activities, such as playing Jenga [63] or completing a button-pressing task [25] or joint n-back task [64].

### The dynamic neural process of interpersonal touch

This study extended the resting-state experimental design to the general population and demonstrated its sensitivity in a naturalistic task, instead of calculating the retest reliability of EEG machines in a laboratory task [65, 66]. Here, we aimed to investigate the pattern by which interpersonal touch alleviates stress and changes the brain-to-brain synchrony in subjects over time. The present dIFC results re-emphasized that brain activity is still dynamic in the resting-state task. This finding is consistent with the results of previous resting-state studies that used fMRI, fNIRS or EEG [67–70]. This finding may also indicate issues with the traditional resting-state baseline measurements: if we segmented and sliced resting-state data and averaged it according to one piece, as we do when measuring ERPs, this could cause misunderstandings of brain activity. According to our dIFC results, the process by which interpersonal touch impacts relationships seems to follow a two-stage model. As the visualization of dIFC in Fig. 4 shows, in the first half of the resting state measurements, the brain-to-brain synchrony fluctuated in both sets of pairs; in the second half of the resting state, the dIFC of romantic lovers in the touch condition of romantic lovers rose steadily and then peaked, while that of the strangers decreased. The high temporal resolution of EEG signals could be a possible cause of our results.

In addition to presenting differences over time domain, we also provided different spatial perspectives from our overhead view of the brain-to-brain synchrony from a whole-brain analysis of EEG signals. For example, Long et al., (2021) [31] reported that interpersonal touch between romantic lovers increased brain-to-brain synchrony between the anterior temporal lobe (ATL) of women and the temporoparietal junction (TPJ) of men to a greater extent than vocal communication, according to fNIRS data from the frontal, temporal, and parietal cortices both hemispheres via 5 sources and 5 detectors (a total of 13 measurement channels). According to fNIRS data on brain activity in the right frontal cortex, Pan et al., (2017) [25] found that the brain-to-brain synchrony between romantic lovers increased significantly in the right superior frontal cortex compared with that of friends and strangers’ frontoparietal regions. The current study covered the entire brain with 29 electrodes (except 1 EOG and 2 reference electrodes: M, left mastoid and M2, right mastoid) based on the international 10–20 labeling system. We used the significant region as seeds to calculate differences in brain-to-brain synchrony between romantic lovers and strangers. Due to the nature of EEG data collection, we selected ROIs with significant differences via whole-brain activity. We chose our ROIs data-driven rather than a priori ROIs choice like the fNIRS study.

Meanwhile, ANOVA assumes that each channel is independent of the other. We also applied SVM, a nonlinear classifier model, to compare differences between romantic lovers and strangers at the whole-brain level in two stages. Furthermore, our best model suggested that nonlinear fits better with our data as the model has the best performance when the kernel is ‘rbf’. However, it is worth mentioning that channel-wise analysis is not equal to studying from a spatial perspective; making a direct comparison between EEG studies and fNIRS studies until source analysis is applied to the higher density of EEG Electrodes is meaningless.

This research differentiated the effects of interpersonal touch in different types of relationships. Romantic lovers may feel relaxed and synchronous while touching, while strangers may feel embarrassed. Similar results were found between voltage magnitude and dIFC. Our result may suggest that interpersonal touch is a double-edged sword. While interpersonal touch between romantic lovers typically increases their well-being and deepens social connection, touch between opposite-sex strangers seems to produce discomfort, resulting in social distancing. Two previous studies showed that while experiencing sensory or psychological pain, a touch from a friend or partner may be more effective in reducing that pain or increasing brain-to-brain synchrony than a touch from a stranger [3, 30]. However, it remains unclear whether the sense of touch is a positive stimulus or a stressor for strangers. Subjects exposed to heat stimuli or electric shock while holding hands with a stranger did not report lower levels of pain or unpleasantness than when alone [71]. Another two studies showed that holding hands with a stranger increased participants’ heart rate [72, 73]. However, other studies have observed a positive effect of interpersonal touch from strangers during exposure to stress [74, 75]. Our findings may provide the evidence that interpersonal touch is a stressor to the strangers which takes up more cognitive resources to adopt this stressful situaction.

### Limitations and future research

Besides, it is important to note the limitations of this study. First, we used EEG-based hyperscanning in this study rather than fNIRS. The ROIs in this study could be used as near-infrared spectral imaging (fNIRS) templates to detect changes in blood oxygen rather than electrical activation. Although fNIRS has reduced temporal accuracy, its spatial accuracy is much higher than electrical activation, which could help us target specific brain regions. In addition, we used AAR [53, 54] to calculate and remove motion artifacts, although such artifacts could not be filtered out completely. FNIRS is less sensitive to motor artifacts, such as head movements, during a 4-minute resting state. Furthermore, we could collect participants’ subjective emotional activation via a questionnaire, such as the Positive and Negative Affect Scale (PANAS), after the end of each block [76–78].

Second, we compared the effects of interpersonal touch between romantic lovers and strangers. However, measuring these two extremes leaves out an intermediate relationship: that of friends. Long’s (2021) [31] study compared brain-to-brain synchrony during interpersonal touch between heterosexual romantic lovers and opposite-sex friends. Other studies examined the effect of a sense of curiosity on friends or acquaintances. For example, one fMRI study included eight pairs of close female friends [79], and a behavior study included several pairs of opposite-sex friends [24]. The concept of friend does not have an objective operational definition like that of the stranger, which usually refers to people who do not know each other or that of romantic lovers, who have an authentic relationship. In addition, the differences between opposite-sex friends, opposite-sex romantic lovers, and potential romantic interests are still ambiguous. Given this ambiguity, we did not include friends in the current study. In future studies, we may include friendships with a clear operational definition and use multiple objective indicators to control the degree of friendship.

Third, we measured the relationships between opposite-sex pairs. As part of our inclusion criteria, we recruited heterosexual romantic lovers with only one current partner and randomly matched sets of male and female strangers to participate in our experiment. A previous study [80] found that women were more inclined to enjoy interpersonal touch than men, while another study [81] found a negative effect of accidental interpersonal touch (AIT) from a male stranger for both men (same-sex touch) and women (opposite-sex touch). Pan et al., (2017) [25] reported a Granger causality analysis and suggested different roles for females and males during cooperation in opposite-sex paired strangers. Previous studies have shown that there might be a sex difference between strangers. In future studies, we may further include same-sex and opposite-sex interpersonal touch conditions to explore the pattern of interpersonal touch by strangers.

In addition, we found that most studies in interpersonal touch focused on pain relief [15, 82, 83]. In discussing the results, we suggest that interpersonal touch between romantic lovers may contribute to stress relief. This inference is based on the observed increase in brain-to-brain synchrony (BtBS) during handholding among romantic partners. Previous studies have demonstrated a link between enhanced BtBS and improved emotional regulation and stress reduction, particularly within intimate relationships. The higher BtBS observed in our study among romantic lovers aligns with these findings, indicating that similar neural mechanisms of emotional regulation could be involved.

However, it is crucial to recognize that our study did not directly measure stress relief. The inference regarding stress reduction is drawn from the observed neural synchrony patterns and their established associations in the literature, rather than direct evidence from our data. Future research should incorporate direct assessments of stress-related physiological or psychological indicators to validate this proposed relationship further.

## Conclusion

In summary, we demonstrated that touch effects differ between romantic lovers and opposite-sex strangers. The results of voltage magnitude in the resting state suggested the difference between romantic lovers and strangers, while the results of dIFC supported that the processing of our brain to interpersonal touch is a dynamic process. The result shows that interpersonal touch may enhance the dIFC between romantic lovers while reducing the dIFC between strangers. The study provides novel insights into the neural dynamics of interpersonal touch, specifically how brain-to-brain synchrony varies between romantic lovers and strangers.

## Data Availability

Because personal privacy is involved and some of the subjects do not want their options to be actively disclosed. All data, scripts, and images in the present study are available to require.
